# The potential of autonomous delivery services to increase fast-food consumption

**DOI:** 10.1017/S1368980024002040

**Published:** 2024-11-11

**Authors:** Simone Pettigrew, Leon Booth, Victoria Farrar, Branislava Godic, Rajith Vidanaarachchi, Charles Karl, Jason Thompson

**Affiliations:** 1 The George Institute for Global Health, University of New South Wales, Sydney, NSW, Australia; 2 Department of Psychiatry, Faculty of Medicine, Dentistry, and Health Sciences, University of Melbourne, Melbourne, VIC, Australia; 3 Melbourne School of Design, The University of Melbourne, Melbourne, VIC, Australia; 4 NTRO, Port Melbourne, VIC, Australia

**Keywords:** Online food delivery, Autonomous vehicles, Food availability, Purchase intentions

## Abstract

**Objective:**

Technological innovations in the online food delivery sector include the use of autonomous delivery vehicles. The aim of the present study was to investigate consumers’ intentions to use these services once they are widely available and their motivations for using them to access unhealthy food.

**Design:**

Online survey including a vignette describing a future world where autonomous food deliveries are in common use in both metropolitan and non-metropolitan areas.

**Setting:**

Australia.

**Participants:**

1078 Australians aged 18 years and older, nationally representative by sex, age and location (metropolitan *v*. non-metropolitan residence).

**Results:**

Around half of the sample reported intending to use an autonomous food delivery service at least once per week for fast food (53 %) and/or healthy pre-prepared food (50 %). Almost two-thirds (60 %) intended using autonomous vehicle deliveries to receive groceries. Around one in five (17 %) anticipated an increase in their fast-food intake as a result of access to autonomous delivery services compared with one in two (46 %) expecting others’ total fast-food intake to increase. The most common reason provided for using autonomous food deliveries was increased convenience. More frequent current fast-food ordering, higher socio-economic status, younger age and regional location were significantly associated with an anticipated increase in fast-food consumption.

**Conclusions:**

The emergence of autonomous food delivery systems may bring both benefits and adverse consequences that in combination are likely to constitute a substantial regulatory challenge. Proactive efforts will be required to avoid negative public health nutrition outcomes of this transport evolution.

The quantity and quality of food available for consumption are key factors influencing diet quality^(1)^ and diet-related health outcomes such as obesity and associated non-communicable diseases^(2)^. Food access is therefore a critical consideration in the development and implementation of nutrition policy, including both ensuring the availability of healthy food and placing restrictions on the availability of unhealthy food^(3)^. Foods prepared outside of the home are typically less healthy than foods prepared in the home, and their frequent consumption increases the risk of diet-related diseases^(4–6)^. Public health nutrition policies focused on consumers’ access to foods prepared outside the home are thus an important component of the regulatory mix.

In recent years, online food delivery systems (OFDS) have dramatically altered the food environment in many countries by enabling rapid access to a wide range of food products prepared outside the home^(7)^. Access is enhanced through two primary mechanisms – increased geographic coverage of individual outlets and the advent of ‘dark kitchens’ that prepare food only for delivery purposes (i.e. they have no public-facing service function)^(7,8)^. Unhealthy options typically dominate the offerings available on OFDS^(9–11)^. Many food-related policies currently in place, such as restaurant zoning restrictions and nutrition labelling requirements, do not typically apply to OFDS^(12,13)^. Continuing strong growth of this sector is predicted^(14)^, which has the potential to increase intake of unhealthy foods and deepen the reliance on pre-prepared foods with an associated loss of cooking skills^(15)^. In combination, these factors are resulting in growing concerns about the implications of the rapid growth in OFDS for diets at the population level, with increasing calls for the development and implementation of public policies specifically designed to limit the negative effects of OFDS^(8,12,16)^.

Other adverse outcomes resulting from OFDS include the hazardous working conditions and inadequate incomes of delivery couriers, increases in traffic congestion associated with deliveries and greater volumes of packaging waste^(12,17,18)^. With many OFDS business models predicated on the basis of avoiding costs through skirting existing employment laws and worker protections, labour issues alone are forecast to make the sector ultimately unsustainable^(17,19)^. Considering the broad range of potential negative impacts, there are concerns that the evolution of OFDS will further stymie the achievement of the UN Sustainable Development Goals^(18)^.

Despite the various negative consequences of OFDS, some forms of home food delivery can also improve access to healthy food and therefore have the potential to constitute a positive influence on diets at the population level^(8)^. For example, the use of grocery home delivery services has been growing rapidly, fuelled in part by the Covid-19 pandemic, and is expected to grow in size by >10 % per annum between 2024 and 2028^(14)^. Studies have shown that compared with in-store shopping, online grocery shopping can result in purchasing larger quantities of nutrient-dense products and smaller amounts of confectionary^(20,21)^. The net effect of OFDS on people’s diets will thus reflect the extent to which these services are used to access a balance of healthy food products *v*. unhealthy on-demand meals and snacks.

The rapid growth of OFDS and the need to attain massive scale to achieve profitability in this low-margin sector are stimulating operational innovations, including the use of autonomous vehicles for the delivery function^(22)^. Autonomous (or self-driving) vehicles have the potential to bring social and economic benefits in the form of fewer crashes per distance travelled, lower greenhouse gas emissions and reduced labour costs for deliveries^(23)^. They exist in numerous forms including cars, vans, shuttles, trucks, buses, trains, trams, drones and sidewalk bots^(24)^. Autonomous vehicle trials are occurring globally across various use cases, including food delivery^(8,25,26)^. It is estimated that autonomous vehicles will dominate road transport systems by 2050^(27)^.

Given the role of food availability in determining food intake^(2,3)^, there are concerns that diet quality could be reduced at the population level due to increased access to unhealthy food through the further scaling up of OFDS made possible by the use of autonomous delivery vehicles, which can increase the speed and geographical range of services, reduce costs and overcome worker shortages during periods of peak demand^(28,29)^. Very little work to date has attempted to assess consumers’ receptiveness to autonomous home delivery services, with the limited available evidence suggesting that around half of the general public may be comfortable with this delivery method^(30,31)^. The primary motivating factors were reported to be increased convenience and expected lower delivery costs resulting from automated delivery processes while inhibiting factors related to issues associated with OFDS in general: enjoyment of browsing in-store and logistical issues associated with being home at the time of delivery^(31)^.

The aim of the present study was to extend this nascent area of research by investigating consumers’ intentions to use autonomous home delivery services to access both healthy and unhealthy foods once they are widely available. Of particular interest was the extent to which people plan to use such services to access unhealthy foods due to the adverse health implications of increased access to these types of products^(8)^. In addition, motivations for using such services to access unhealthy food were explored to provide insights into the strategies that may be needed to reduce potential harms.

## Methods

This study was part of a larger project investigating the social implications of the emergence of autonomous vehicles^(32)^. Among a broader set of transport issues, Australian adults’ perceptions of autonomous food delivery services were explored via a national online survey. In November–December 2022, Pureprofile, an ISO-accredited social research agency, applied quotas to recruit a nationally representative sample on the demographic attributes of sex, age and residential location (metropolitan *v*. non-metropolitan area, identified on the basis of postcode^(33)^). A total of 1078 Australian adults completed the survey (the survey sample profile is shown in Table 1). All participants provided informed consent, and the study was approved by a University Human Research Ethics Committee.

**Table 1.** Survey sample profile

Characteristic	Total (*n* 1078^*^)		Males (*n* 534)		Females (*n* 541)	
	*n*	%	*n*	%	*n*	%
Age (years)						
18–34	315	29	148	28	166	31
35–54	357	33	182	34	174	32
55+	406	38	204	38	201	37
Socio-economic status^†^						
Low (deciles 1–4)	353	33	168	32	184	34
Medium (deciles 5–7)	337	31	163	31	173	32
High (deciles 8–10)	388	36	203	38	184	34
Location (metropolitan)	736	68	379	71	355	66
Av. frequency of fast-food consumption per week						
Never	129	12	65	12	64	12
<3 times per week	875	81	432	81	441	82
3+ times per week	74	7	37	7	36	7
Meets fruit intake guideline	521	48	252	47	267	49
Meets vegetable intake guideline	73	7	30	6	43	8

* Two respondents identified as non-binary and one respondent elected to not respond to the sex question.

† Derived from residential postcode using the Australian Bureau of Statistics’ Socio-Economic Indexes for Areas classification^(33)^.

The survey items relevant to the present study assessed current consumption of fast food and methods of fast-food delivery, anticipated own and others’ use of autonomous food deliveries once they are available and perceived reasons for increases in own and others’ fast-food consumption once autonomous delivery systems are common. Current behaviours were assessed prior to exposure to a detailed vignette describing how autonomous forms of transport, including food deliveries, are expected to exist in the future. The use of such vignettes in autonomous vehicle research is recommended as an effective form of stimulating responses on a topic about which few consumers are likely to have given prior consideration^(27)^. The questions relating to the anticipated future use of autonomous food delivery services were asked after vignette exposure. Other survey items assessed transport behaviours and alcohol consumption, the results of which have been reported elsewhere^(34,35)^.

**Figure 1.**
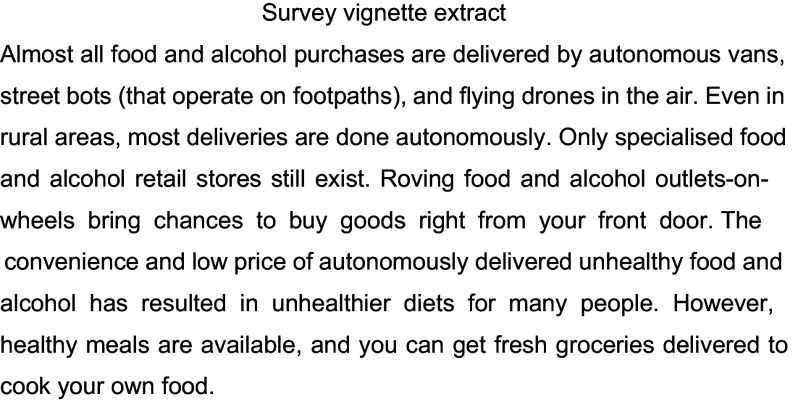
Survey vignette content pertaining to food delivery.

The content of the vignette was based on previous research involving fifty-two experts representing a range of relevant sectors (e.g. public health, transport, urban planning and telecommunications) who described their expectations for the progressive roll-out of autonomous vehicles and the implications for a wide range of lifestyle behaviours^(28)^. An extract of the vignette showing the content relevant to the present study is shown in Fig. 1, and the full vignette is provided in the supplementary materials. The vignette content intentionally included a strong emphasis on food and drink deliveries to stimulate contemplation of how access to these product categories would change in an autonomous future.

Current consumption of fast food was assessed in the survey as follows: ‘In an average week, how often do you eat (eat in or take out) an unhealthy meal from fast-food places such as eating burgers, fried chicken, pizza, burritos and other unhealthy meals’, with five response options ranging from ‘Never’ to ‘5 or more times per week’. Methods of obtaining fast food were identified by asking ‘What method would you usually use to receive the fast food?’, with take-away, eat-in and delivery response options provided. Delivery options included ‘Have the fast food delivered by the food producer’ and ‘Have the fast food delivered by app-based food delivery services (Uber Eats, Menulog, DoorDash, Deliveroo, Milkrun, etc.)’. Order frequency was assessed via eight response options ranging from ‘Never’ to ‘Every day’. As a measure of current diet quality, respondents reported their current daily intake of fruit and vegetables.

Anticipated own use of autonomous food deliveries was assessed by asking three questions after exposure to the vignette: ‘In this world, how often do you think you would use autonomous delivery services to get fast food?’, ‘In this world, how often do you think you would use autonomous delivery services to get your groceries?’, and ‘In this world, how often do you think you would use autonomous delivery services to get healthy pre-prepared food options (e.g. salads, sushi, rice paper rolls, wraps)?’, each with five response options ranging from ‘Never’ to ‘7+ times per week’.

Changes in own and others’ fast-food consumption were measured by asking ‘If you were living in this world, would the frequency of your unhealthy fast-food consumption change? Examples of unhealthy fast foods include eat-in or take-out burgers, fried chicken, pizza and burritos?’ and ‘In this world, how would you expect most people’s consumption of unhealthy fast food to change (if at all)?’, with possible responses including ‘Decrease’, ‘Stay the same’ and ‘Increase’ options. Expected reasons for own and others’ changes in fast-food consumption were examined by asking ‘Can you please describe why your fast-food consumption might change?’ and ‘Can you please describe why other people’s fast-food consumption might change?’, with response options including ‘Cheaper price’, ‘Faster delivery’, ‘More convenient’, ‘Easier than cooking’ and ‘Faster than preparing other food’. Multiple reasons could be selected.

Descriptive analyses were conducted to assess the percentage of respondents selecting varying response options, with *χ*^2^ analyses and McNemar’s tests conducted to identify any significant differences in proportions. Compliance with fruit and vegetable consumption guidelines was deemed to have occurred when respondents reported consuming at least two servings of fruit per d and five servings of vegetables per d, respectively^(36)^. An alpha level of *P* < .001 was applied to account for the number of comparisons.

Binary logistic generalised linear models were used to identify factors associated with respondents’ anticipated (i) increase in fast-food consumption, (ii) use of autonomous fast-food delivery services, (iii) use of autonomous grocery delivery services and (iv) use of autonomous healthy pre-prepared food delivery services. The following independent variables were entered into the model: age, sex (male *v*. female), location (metropolitan *v*. regional), socio-economic status decile, frequency of fast-food ordering and healthy diet composite score (0 = compliance with neither fruit nor vegetable guideline, 1 = compliance with fruit or vegetable guideline, 2 = compliance with both guidelines). Respondents identifying as non-binary or who did not wish to disclose their gender were excluded from the model analyses due to the small subsample size (*n* 3). To allow for comparisons of effect sizes between the independent variables, the regression coefficients resulting from the model were partially standardised according to the independent variables’ units of measurement and converted into OR. This was achieved by multiplying the unstandardised coefficient for each independent variable by its sd^(37)^.

## Results

In response to the vignette describing a future where autonomous food deliveries are widely available, around half of the respondents reported intending to use this form of delivery at least once per week to access fast food (53 %) and/or healthy pre-prepared food (50 %). Almost two-thirds (60 %) intended using autonomous vehicle deliveries to receive groceries (see Table 2).

**Table 2.** Anticipated frequency of various forms of food delivery via autonomous vehicles (*n* 1078)

	Never %	1–2 times per week %	3+ times per week %
Fast food	47^a^	45^ab^	8^a^
Healthy pre-prepared options	50^a^	42^a^	9^a^
Groceries	40^b^	50^b^	10^a^

Note: Proportions within columns with different superscripts are significantly different from each other at *P* < 0.001.

While most respondents expected that their own intake of fast food would not change in the scenario depicted in the vignette (72 %), almost one in five (17 %) anticipated an increase (see Table 3). By comparison, around half (46 %) expected others’ fast-food intake to increase as a result of access to fast food via autonomous vehicle deliveries.

**Table 3.** Anticipated changes in fast-food consumption resulting from access to autonomous vehicle food deliveries (*n* 1078)

	Decrease %	No change %	Increase %
Own fast-food consumption	11^a^	72^a^	17^a^
Others’ fast-food consumption	5^b^	60^b^	46^b^

Note: Proportions within columns with different superscripts are significantly different from each other at *P* < 0.001.

Expected reasons for increases in fast-food consumption once autonomous deliveries are available were similar for self and others (see Table 4). Greater convenience (69 % self, 79 % others) and faster delivery (60 % self, 62 % others) were the most frequently nominated reasons, followed by the reduction in effort required for cooking (46 % self, 57 % others) and the time involved in food preparation (43 % self, 46 % others). Lower cost was least frequently nominated but selected by sizable minorities (36 % self, 23 % others).

**Table 4.** Perceived reasons for increases in own and others’ consumption of fast food resulting from access via autonomous deliveries

	Own consumption (*n* 179)		Others’ consumption (*n* 492)	
	*n*	%^*^	*n*	%^*^
More convenient	124	69^a^	390	79^a^
Faster delivery	108	60^ab^	304	62^b^
Easier than cooking	82	46^bc^	281	57^b^
Faster than preparing other food	77	43^c^	224	46^c^
Cheaper price	65	36^c^	114	23^d^

* Percentage of those anticipating an increase in consumption.

Note: Proportions within columns with different superscripts are significantly different from each other at *P* < 0.001.

In effect size order (as per the standardised OR), the regression analysis identified more frequent current fast-food ordering (OR 1·40 (1·17, 1·66), *P* <.001)), higher socio-economic status (OR 1·36 (1·13, 1·64), *P* = .001) and regional location (OR 1·23 (1·03, 1·47), *P* = .022) as being significantly associated with an anticipated increase in fast-food consumption once autonomous deliveries are available (see Table 5). Older respondents were less likely than younger respondents to expect their fast-food consumption to increase (OR .70 (.58, .85), *P* = <.001)). Sex and current compliance with fruit and vegetable intake guidelines were not found to be significant predictors in the model.

**Table 5.** Generalised linear model of factors associated with anticipating increased own fast-food consumption

Factor	B	95 % CI	OR	95 % CI	Standardised OR	95 % CI	*P*-value
Age	–0·02	–0·03, –0·01	0·98	0·97, 0·99	0·70	0·58, 0·85	**<0·001**
Sex							
Female	<0·01	–0·33, 0·33	1·00	0·72, 1·4	1·00	0·85, 1·18	0·993
Male^*^	–		–		–		–
Location							
Regional	0·44	0·06, 0·82	1·56	1·07, 2·27	1·23	1·03, 1·47	**0·022**
Metro^*^	–		–		–		–
Socio-economic status (decile)	0·11	0·04, 0·18	1·12	1·04, 1·19	1·36	1·13, 1·64	**0·001**
Frequency of fast-food ordering	0·19	0·09, 0·29	1·21	1·1, 1·34	1·40	1·17, 1·66	**<0·001**
Compliance with fruit and vegetable intake guidelines^†^	<0·01	–0·28, 0·28	1·00	0·76, 1·33	1·00	0·85, 1·19	0·976

* Reference category.

† At least two servings of fruit per d and five servings of vegetables per d^(36)^.

Similar factors were found to be significantly associated with intentions to use autonomous delivery services to access fast food, groceries and pre-prepared healthy meals (see Table 6). For all three forms of food delivery, the current frequency of fast-food ordering (OR 1·75–2·20) and younger age (OR 0·52–0·81) predicted intentions. Higher socio-economic status was associated with intending to use autonomous deliveries for fast food (OR 1·18) and healthy meals (OR 1·18), but not groceries. Compliance with fruit and vegetable intake guidelines was only associated with intentions relating to healthy meal deliveries (OR 1·17).

**Table 6.** Generalised linear models of factors associated with food consumption and autonomous delivery options

	Intended use of autonomous fast-food delivery services				Intended use of autonomous grocery delivery services				Intended use of autonomous healthy meal delivery services			
Factor	B	95 % CI	Standardised OR	95 % CI	B	95 % CI	Standardised OR	95 % CI	B	95 % CI	Standardised OR	95 % CI
Age	–0·04***	–0·05, –0·03	0·52***	0·44, 0·61	–0·01**	–0·02, –0·01	0·81**	0·69, 0·93	–0·03***	–0·04, –0·02	0·62***	0·54, 0·71
Sex												
Female	0·06	–0·22, 0·34	1·19	0·51, 2·78	0·01	–0·25, 0·27	1·03	0·47, 2·24	0·10	–0·16, 0·37	1·37	0·62, 3·03
Male^†^	–		–		–		–		–		–	
Location												
Regional	0·16	–0·16, 0·48	1·08	0·93, 1·25	0·15	–0·15, 0·44	1·07	0·93, 1·23	0·05	–0·25, 0·35	1·02	0·89, 1·18
Metro^†^	–		–		–		–		–		–	
Socio-economic status (decile)	0·06*	0·01, 0·11	1·18*	1·02, 1·36	0·02	–0·03, 0·07	1·05	0·92, 1·21	0·06*	0·01, 0·11	1·18*	1·03, 1·36
Frequency of fast-food ordering	0·45***	0·36, 0·55	2·20***	1·86, 2·6	0.32	0·23, 0·42	1·75***	1·50, 2·06	0·33***	0·24, 0·42	1·79***	1·53, 2·08
Compliance with fruit and vegetable intake guidelines^‡^	–0·18	–0·41, 0·06	0·90	0·78, 1·04	0·03	–0·19, 0·24	1·02	0·89, 1·16	0·26*	0·04, 0·48	1·17*	1·02, 1·34

B = regression coefficient.

*P < 0.05, **P < 0.01, ***P < 0.001.

† Reference category.

‡ At least two servings of fruit per d and five servings of vegetables per d^(36)^.

## Discussion

Greater availability of unhealthy foods via OFDS is noted in the literature as having the potential to increase intake of these products, thereby compromising public health^(8,12,16)^. The results of the present study indicate that many consumers may recognise that increased accessibility of fast food, in this case via autonomous delivery vehicle services, is likely to result in an overall greater consumption of unhealthy food. In accordance with attribution theory^(38)^, respondents were more likely to consider others to be vulnerable to autonomous fast-food deliveries than themselves. Around one in five anticipated that their own total fast-food intake would be greater as a result of access to autonomous delivery services compared with one in two expecting others’ total intake to increase. At a population level, either of these outcomes would represent substantial growth in fast-food consumption.

A key finding of the present study was that people residing in non-metropolitan areas were more likely than their metropolitan counterparts to anticipate an increase in their fast-food consumption once they had access to autonomous food deliveries. This may be due to expectations of wider area coverage of delivery services resulting from reduced labour costs that might otherwise make such trips economically unviable. Increased access to unhealthy foods in regional areas is of concern given higher rates of obesity among those living outside of metropolitan areas^(39)^.

Similar to the results of prior studies examining factors associated with using OFDS^(8,40–42)^, other characteristics associated with the anticipated use of autonomous delivery services once they are widely available were more frequent current consumption of fast food, higher socio-economic status and younger age. These characteristics may therefore signal important target groups for interventions designed to promote healthier food choices among those using OFDS both in the present and once autonomous delivery services are commonplace. Specifically in terms of fast-food deliveries, the lack of association with respondent sex (found to be significant in some prior work^(40,41)^) and compliance with fruit and vegetable guidelines (an indicator of diet quality) could mean that increasing access to fast food via autonomous deliveries may result in a wider subsection of the population consuming fast food on a regular basis.

A potential positive outcome of the present study was that somewhat more respondents anticipated using autonomous food delivery services to regularly access groceries (60 %) compared with the proportion intending to use them to regularly access fast food (53 %) and half expected to use autonomous deliveries to receive healthy pre-prepared food options (50 %). This is somewhat encouraging in the light of research demonstrating that online grocery orders tend to be healthier overall than in-store purchases^(20,21)^. However, while access to healthy foods/groceries via autonomous deliveries represents a favourable outcome for those with mobility limitations^(28)^, food shopping can be an important source of physical activity, and as such, an increased reliance on delivery services could have detrimental impacts on overall activity levels^(43,44)^.

The dominance of convenience as a stated reason for one’s own and others’ use of autonomous food delivery services in the present study is aligned with previous research highlighting the primary role of convenience as a motivation for using OFDS^(8,17,30,45)^. It is also consistent with technology acceptance frameworks that emphasise the importance of consumers’ perceptions of the usefulness and ease of use of new technologies in determining adoption levels^(46,47)^. The vignette presented to respondents in the survey described autonomous food deliveries as convenient and inexpensive; the study results indicate that the former attribute was considered by respondents to be substantially more important than the latter. The lesser perceived relevance of cost ascribed to both own and others’ intentions could be partially attributed to the already low delivery charges typically applied by OFDS^(17)^.

The similarities noted above in terms of general OFDS motivations described in the literature and those identified in the present study pertaining to autonomous vehicle deliveries are mirrored in recent qualitative research examining Australians’ attitudes to autonomous food and beverage deliveries^(31)^. The latter found that few participants were specifically concerned about the type of vehicle making the delivery, and instead, the primary focus was on speed, cost and food temperature upon arrival. While this emphasis on generic rather than transport-method-specific outcomes may to some extent reflect a lack of familiarity with autonomous delivery options and therefore an inability to discuss them in detail, it may also be the result of consumers already having access to multiple forms of food delivery and autonomous vehicles being perceived as just an additional alternative.

### Policy implications

There are two competing aspects to consider in developing policies to address the emergence of autonomous food delivery services. First, autonomous vehicles are forecast to greatly reduce the mortality and morbidity associated with vehicle crashes globally^(48)^. Specifically in terms of product deliveries, they may reduce, and ultimately eradicate, the need for humans to engage in this often unhealthy and dangerous occupation^(49)^. It is therefore envisaged that autonomous vehicles will be a critically important component of future transport systems^(50)^. Second, the results of the present study indicate that autonomous food delivery services have the potential to increase obesity and other nutrition-related diseases by enhancing the availability of unhealthy food. Relevant policies therefore need to ensure that the reductions in road trauma that are expected to accompany the introduction of autonomous vehicles do not come at the expense of exposure to other population-level public health risks such as poor nutrition and physical inactivity.

Despite the nascent status of autonomous food delivery systems, it is critical for governments to take proactive steps to shape future developments in this sector. As evidenced by the introduction of transport innovations such as Uber, industry and technological ‘disruptions’ can occur too quickly for governments to be able to regulate effectively once a level of market penetration has been achieved^(51)^. In the case of OFDS, there are concerns that the highly concentrated industry has grown so quickly that any attempts to introduce restrictions that increase operating costs or reduce consumer appeal would be vigorously opposed by both the companies and their customers^(8)^. Early action before autonomous delivery options become mainstream is therefore vital.

A further barrier to the development and implementation of relevant policies relating to autonomous deliveries is that OFDS already fall within a regulatory grey zone due to a lack of application of many existing food retail requirements^(8,13)^. This means that current regulatory loopholes need to be addressed for OFDS in general, as well as considering the longer-term implications of autonomous delivery systems. For example, there are calls for food labelling policies that apply to products sold in supermarkets to also apply to items available for sale on OFDS, such as salient nutrition labelling in both detailed and summary formats (examples being mandatory nutrition information panels and the voluntary Health Star Rating system in use in Australia)^(16,28)^. Other recommendations include setting quotas for the proportion of healthy menu items, implementing choice architecture strategies that involve making healthy options the default and featured offerings and applying additional taxes to home-delivered unhealthy foods^(8,16,28,42)^. Such strategies could improve the healthiness of home-delivered food, regardless of the method of delivery. This is important in the context of around half of the present sample indicating they would use autonomous deliveries to access healthy foods, making it essential for consumers to be able to identify healthy options while shopping.

Other strategies that could apply specifically to autonomous deliveries could include banning some forms of transport (e.g. street bots that can congest footpaths and impede pedestrians) and some delivery locations (e.g. schools)^(28)^. Finally, while it has been proposed that OFD companies should be required to collect and use consumer ordering and financial data responsibly^(16)^, the use of cameras to guide autonomous delivery vehicles raises additional privacy and data access issues relating to how video footage of customers, their families and their neighbours is accumulated and managed. This issue requires prompt and comprehensive attention.

### Study strengths and limitations

The present study appears to be one of the first to assess consumers’ intentions to use autonomous delivery vehicles to access food and the potential for increased consumption of fast food resulting from enhanced availability. It therefore provides important initial evidence supporting the need to proactively implement strategies to ameliorate the potential effects of this emerging form of unhealthy food access.

The primary limitation of the present study was the use of a scenario-based vignette that depicted a single potential future. This approach is likely to have had a priming effect resulting from the specification of particular diet-related outcomes associated with the wide availability of autonomous food delivery services. However, the vignette appeared to be effective in stimulating respondents to contemplate alternative futures, which was evidenced by varying reported views within the sample on the likelihood of changes in their own and others’ consumption of unhealthy food. A second limitation was the confinement of data collection to a single country. Future research could administer a wider range of potential scenarios across a broader group of countries. Third, a non-probability web panel was used for respondent recruitment. The use of demographic quotas ensured a roughly representative national sample, but it is possible that the sample was skewed on unassessed relevant psychographic characteristics (e.g. novelty seeking). Finally, the requirement to complete an online survey assumed a level of computer access and proficiency that would have excluded some potential respondents. Future work in this area could consider alternative forms of participant recruitment.

In conclusion, the emergence of autonomous food delivery systems is likely to bring both benefits and adverse impacts that in combination constitute a substantial regulatory challenge. The complexity of this challenge makes it essential for proactive consideration to be given to optimal methods of ensuring autonomous food delivery systems result in improved, not exacerbated, public health nutrition.

## Supporting information

Pettigrew et al. supplementary material Pettigrew et al. supplementary material

## Supplementary material

For supplementary material accompanying this paper, visit https://doi.org/10.1017/S1368980024002040.
