# Supplementary material for: The potential of autonomous delivery services to increase fast-food consumption
Source: Public Health Nutr. 2024 Nov 11;27(1):e246. doi: 10.1017/S1368980024002040 (PMC11705010; doi:10.1017/S1368980024002040)
Supplement: Pettigrew et al. supplementary material [file S1368980024002040sup001.docx]

**Supplementary materials**

**Survey vignette – full scenario**

Imagine a world where you are no longer allowed to drive and all vehicles on public roads operate autonomously without drivers. These autonomous vehicles communicate with each other and centralised computer systems, which allows them to operate swiftly and safely.

There are four autonomous vehicle options available to you: You can own a personal autonomous vehicle, which is a highly convenient but expensive option. You can use ride-hail autonomous vehicles (like a self-driving taxi/uber), which is a cheaper way to get around than owning your own autonomous vehicle and is convenient. You can use autonomous public transport options that are very cheap and reliable. For example, autonomous shuttle buses are available that pickup/drop off people who are going in a similar direction. You can buy or hire a personal automated flying vehicle that is the most expensive option but typically the fastest way to travel moderate distances. Footpaths and cycleways are everywhere, making it easier to walk, cycle, and scoot to destinations. It is also very safe to travel this way because autonomous vehicles are highly effective at avoiding collisions.

Almost all food and alcohol purchases are delivered by autonomous vans, street bots (that operate on footpaths), and flying drones in the air. Even in rural areas, most deliveries are done autonomously. Only specialised food and alcohol retail stores still exist. Roving food and alcohol outlets-on-wheels bring chances to buy goods right from your front door.

The convenience and low price of autonomously delivered unhealthy food and alcohol has resulted in unhealthier diets for many people. However, healthy meals are available, and you can get fresh groceries delivered to cook your own food.
